# Prognosis of patients without perfusion defects with and without rest study in myocardial perfusion scintigraphy

**DOI:** 10.1186/2191-219X-3-58

**Published:** 2013-07-31

**Authors:** Lars Edenbrandt, Mattias Ohlsson, Elin Trägårdh

**Affiliations:** 1 Clinical Physiology and Nuclear Medicine Unit, Lund University, Skåne University Hospital, Malmö, Sweden; 2 Computational Biology and Biological Physics, Lund University, Lund, Sweden

**Keywords:** Myocardial perfusion imaging, Ischemic heart disease, Prognostic value

## Abstract

**Background:**

Stress myocardial perfusion scintigraphy (MPS) is widely regarded as a useful imaging modality for diagnosing patients with suspected ischemic heart disease. Current European guidelines recommend stress study to be performed first since rest study can be omitted if stress study is interpreted as normal. Thus, a rest study should only be performed in patients with equivocal or abnormal studies. The aim of the present study was to investigate the prognosis of a normal stress-only MPS compared to a normal stress-rest MPS in a retrospective manner and also with regard to normal/abnormal left ventricular function data.

**Methods:**

All 4,820 patients who underwent ^99m^Tc MPS at Skåne University Hospital in Malmö, Sweden, in 2004 to 2007, for suspected or management of known ischemic heart disease were considered. The physician in clinical charge of the investigation decided whether a rest study was necessary or not. Based on the final report according to clinical routine, only patients with a normal perfusion study (no infarction or inducible ischemia) were included. The endpoints were non-fatal acute coronary syndrome or death from ischemic cardiac origin.

**Results:**

A total of 3,426 patients with a normal perfusion study were included. Of these, 2,215 patients had a stress-only study and 1,211 patients had both stress and rest studies. Mean follow-up was 6.2 years. The lowest event rate was found in the normal stress-only group (0.56% for normal stress-only patients vs. 1.42% for normal stress-rest patients; *p* < 0.0001). When dividing patients according to sex and stress type, the best prognosis was also found in the normal stress-only group (*p* < 0.0001 for all comparisons). Regarding left ventricular function data, we did not find any significant difference in event rate between normal vs. abnormal ejection fraction (EF), normal vs. abnormal end-diastolic volume (EDV) or normal EF, and EDV vs. abnormal EF or EDV for either the normal stress-only patients or the normal stress-rest patients.

**Conclusions:**

Patients with a normal stress-only study had an excellent prognosis over a mean follow-up time of 6 years. Thus, omitting the rest study if the stress study is normal is a safe procedure.

## Background

Stress single-photon emission computed tomography (SPECT) myocardial perfusion scintigraphy (MPS) with technetium-99 m (^99m^Tc) is widely regarded as a clinically useful non-invasive imaging modality for diagnosing patients with suspected ischemic heart disease and assessing patient risk [1-3]. Current MPS imaging guidelines differ in their opinion whether a rest study is necessary or not when stress study is interpreted as normal. The European Association of Nuclear Medicine and European Society of Cardiology recommend that the stress study should be performed first [4] since the rest study can be omitted if the stress study is interpreted as normal, whereas US guidelines state that issues regarding the imaging sequence (stress vs. rest first) are not fully settled [5].

The advantages of using the stress-only approach when the stress study is interpreted as normal are reduced radiation exposure, lowered costs by eliminating unnecessary imaging time and radiopharmacologic doses, and improved efficiency in the nuclear medicine department by freeing up camera time. Ueyama et al. [6] recently found that patients determined as having a normal SPECT on the basis of stress imaging alone have a similar cardiac event rate as those who have a normal SPECT on the basis of evaluation of both stress and rest images. They included 1,125 patients in their study with a mean follow-up time of 3.4 years. Chang et al. [7] made the same conclusion in a major study including 16,854 patients. None of these studies have included patients with abnormal left ventricular (LV) function data.

The main aim of the present study was to investigate the prognosis of a normal stress-only MPS compared to a normal stress-rest MPS in a retrospective manner, with non-fatal acute coronary syndrome or death from ischemic cardiac origin as end points. The second aim was to evaluate differences in prognosis for patients with normal/abnormal LV function data in the different study protocol groups.

## Methods

### Study population and interpretation of studies

Patients who underwent MPS for clinically indicated reasons at Skåne University Hospital, Malmö, Sweden between 1 January 2004 and 31 December 2007 were considered for inclusion. Patients <20 years of age were excluded.

Patients were divided into subgroups based on the final report according to clinical routine. The physicians responsible for the studies interpreted the images on the basis of integration of the rotating raw projection data, tomographic perfusion images, gated SPECT information, quantitative perfusion SPECT results, and a computer-assisted interpretation made by EXINI Heart™ (EXINI Diagnostics AB, Lund, Sweden). A stress study interpreted as normal (perfusion assessed to be homogeneous throughout the myocardium) by the nuclear medicine physician in charge was not followed by a rest study.

The following analyses were performed:

1. First, all patients regarded as ‘normal’ with regard to perfusion (i.e., clearly stating ‘no ischemia’, and ‘no infarction’ in the conclusion section of the report) were divided into two groups based on imaging protocol (stress-only or stress-rest). All other patients were considered ‘non-normal’ (abnormal or equivocal). For this analysis, all patients were included, regardless of LV function data.

2. In the next analyses, patients were also compared with regard to sex (male, female) and stress type (pharmacologic, i.e., adenosine or dobutamine, exercise test). Also in these analyses, all patients with normal perfusion were included, regardless of LV function data.

3. In the final analysis, normal stress-only and normal stress-rest patients were divided into groups according to end-diastolic volume (EDV) and ejection fraction (EF). Three analyses were performed: (1) patients with normal EF vs. abnormal EF, (2) patients with normal EDV vs. abnormal EDV, and (3) patients with normal EF and EDV vs. abnormal EF or EDV. Normal EF was defines as ≥55%, and normal EDV was defined as ≤185 ml for men and ≤135 ml for women. EF and EDV were only reported after September 2004, so all patients before this or all patients with missing data in the final report were excluded.

The study was approved by the local ethics committee at Lund University and follows the Declaration of Helsinki.

### MPS protocol

The MPS studies were performed using a 2-day gated stress/non-gated rest ^99m^Tc-tetrofosmin protocol, starting with injection of 600-MBq ^99m^Tc-tetrofosmin at stress. Patients were stressed using either maximal exercise on a bicycle or pharmacologic test with adenosine or dobutamine. Standard protocol for the exercise test was a ramp test with start of 50 W for men and 30 W for women with increments of 15 W/min for men and 10 W/min for women. The exercise was continued for at least 1 min after the injection of the tracer. The criteria for termination of the exercise test were at least 85% of age-predicted maximal heart rate and at least 17 (of 20) on the rated perceived exertion scale. If the heart rate criterion was not met, the exercise was converted to a pharmacologic test. Standard adenosine protocol was a continuous infusion at 100 μg/kg/min for 1 min and then 140 μg/kg/min for 4 min, with tracer injection after 3 min of infusion. Dobutamine was infused incrementally, starting at a dose of 10 μg/kg/min and increasing at 3-min intervals to 20, 30, and 40 μg/kg/min. If the heart rate did not reach 85% of age-predicted maximal heart rate, atropine was used.

Normal findings at stress were not followed by a rest study. Not normal stress studies were followed by a rest study with injection of 600-MBq ^99m^Tc-tetrofosmin, within 1 week of the stress study. In our clinical routine, the physician in charge evaluates the stress images and decides whether a rest study is necessary or not.

Stress and rest acquisition began about 60 min after the end of the injection of ^99m^Tc-tetrofosmin. Images were obtained according to established clinical protocols, using SPECT over 180° elliptical, autocontour rotations from the 45° right anterior oblique position, with a dual-head gamma camera, e.cam (Siemens AG Medical Solutions, Erlangen, Germany). Patients were imaged in the supine position. Low-energy high-resolution collimator and a zoom factor of 1.0 were used. We obtained 64 (32 views per camera) projections in a 128 × 128 matrix, with an acquisition time of 25 s per projection. Stress images were gated to the electrocardiogram using eight frames per cardiac cycle. No automatic motion-correction program was applied; instead, the acquisition was repeated if motion was detected. Tomographic reconstruction and calculation of short and long axis slice images were performed using e.soft (Siemens AG Medical Solutions). Non-attenuation-corrected images were reconstructed with filtered back projection. A 2D Butterworth prereconstruction filter was used with a cutoff frequency of 0.45, order 5. Attenuation-corrected images were reconstructed with an iterative algorithm, with six iterations [8] where a ramp filter was applied on the error projection prior to back projection. A Butterworth filter with a cutoff frequency of 0.40, order 5, was applied for regularization. Attenuation maps were generated from simultaneous transmission measurement using a ^153^Gd multiple-line source (Siemens AG Medical Solutions) [9]. Attenuation correction was used for all patients.

### Follow-up and outcomes

On 31 December 2010, all study patients were evaluated according to cardiac events. The primary end points were non-fatal acute coronary syndrome (myocardial infarction or unstable angina pectoris) or death from ischemic cardiac origin (myocardial infarction, sudden cardiac death, chronic ischemic myocardial disorder). The Register of Information and Knowledge about Swedish Heart Intensive Care Admissions, which includes all patients admitted to hospitals with coronary care units in Sweden, were used to identify patients who had acute coronary syndromes during the follow-up time after the MPS [10]. Information is reported on case record forms including 100 variables and has been described elsewhere [11,12]. Briefly, the register includes information on age, sex, interventional procedures, and discharge diagnosis. Data on mortality were obtained from the Swedish National Cause of Death Register. Follow-up time and cardiac events were determined for the entire study population.

### Statistical analysis

Kaplan-Meier estimates of the survival function were used together with the log-rank test to indicate significance between the different groups of patients. The date of the MPS study was defined as *T* = 0 in the analysis. Average annual event rates were expressed as the number of patients having an event (acute coronary syndrome or death from ischemic cardiac origin) divided by the total number of patient follow-up years. Statistical analysis was carried out using the R software environment (R Foundation for Statistical Computing, Vienna, Austria) [13].

## Results and discussion

### Results

Normal stress-only patients had a mean age of 61.7 years (range 21 to 95 years, standard deviation (SD) 11.1) and consisted of 2,215 individuals (60.0% females). Normal stress-rest patients had a mean age of 62.1 years (range 21 to 93 years, SD 11.7) and consisted of 1,211 individuals (46.7% females). The remaining patients (non-normal) had a mean age of 64.8 years (range 25 to 93 years, SD 10.6) and consisted of 1,394 individuals (34.0% females). The normal stress-only patients were stressed with adenosine in 35.6%, exercise test in 64.1%, and dobutamine in 0.2%. The normal stress-rest patients were stressed with adenosine in 56.5%, exercise test in 41.9%, and dobutamine in 1.7%. Mean follow-up time was 6.2 years (SD 1.7) with a total number of events of 414 (8.6%).

#### Comparison of event rate with regard to perfusion data

**Figure 1:**
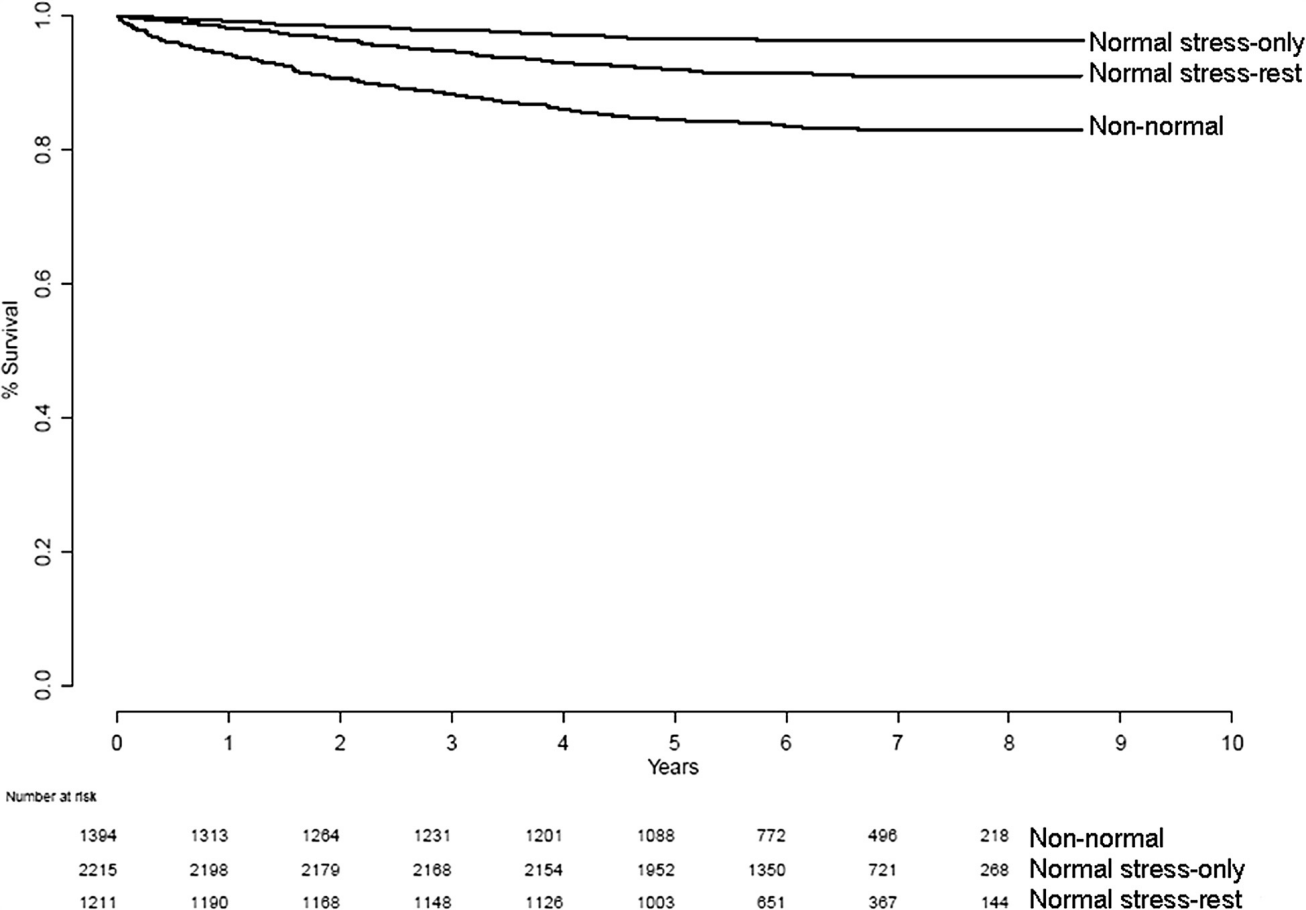
Survival for the entire study population according to MPS protocol.

There was a statistically significant difference in event rate between the three groups, with the lowest event rate in the normal stress-only group, followed by the normal stress-rest group (*p* < 0.0001; Figure 1). Table 1 shows the number of patients, number of events, and annualized event rate for the stress-only and stress-rest groups. For the non-normal group, annualized event rate was 2.78%.

**Table 1:** Number of patients, number of events, and annualized event rate (%) for the different groups

	**Normal stress-only group**			**Normal stress-rest group**		
	**Patients**	**Events**	**Annual event rate**	**Patients**	**Events**	**Annual event rate**
All	2,215	79	0.56	1,211	105	1.42
Normal EF	1,351	41	0.47	754	56	1.28
Abnormal EF	60	1	0.26	83	8	1.58
Normal EDV	1,365	42	0.48	753	56	1.27
Abnormal EDV	46	0	0	84	8	1.64
Normal EF and EDV	1,326	41	0.48	712	51	1.23
Abnormal EF or EDV	85	1	0.18	125	13	1.76
Women	1,329	40	0.47	566	34	0.97
Men	886	39	0.70	645	71	1.82
Pharmacologic stress	795	25	0.53	704	71	1.68
Exercise	1,420	54	0.58	507	34	1.07

#### Comparison of event rate with regard to sex

There were 1,329 women with normal perfusion data in the normal stress-only group compared to 566 in the normal stress-rest group. There was a statistically significant difference in event rate between the groups, with a lower event rate in the normal stress-only group (*p* < 0.0001).

There were 886 men in the normal stress-only group and 645 in the normal stress-rest group. Again, there was a statistically significant difference in event rate between the groups, with the lower event rate in the normal stress-only group (*p* < 0.0001). Table 1 shows the number of patients, number of events, and annualized event rate for the groups.

#### Comparison of event rate with regard to stress type

There were 795 patients in the normal stress-only group who underwent pharmacologic stress (adenosine or dobutamine) and 704 in the normal stress-rest group. We found a statistically significant lower event rate in the normal stress-only group (*p* < 0.0001).

There were 1,420 patients in the normal stress-only group who underwent exercise stress and 507 in the normal stress-rest group. There was a statistically significant lower event rate in the normal stress-only group (*p* < 0.0001). Table 1 shows the number of patients, number of events, and annualized event rate for the groups.

#### Comparison of event rate with regard to LV function data

**Figure 2:**
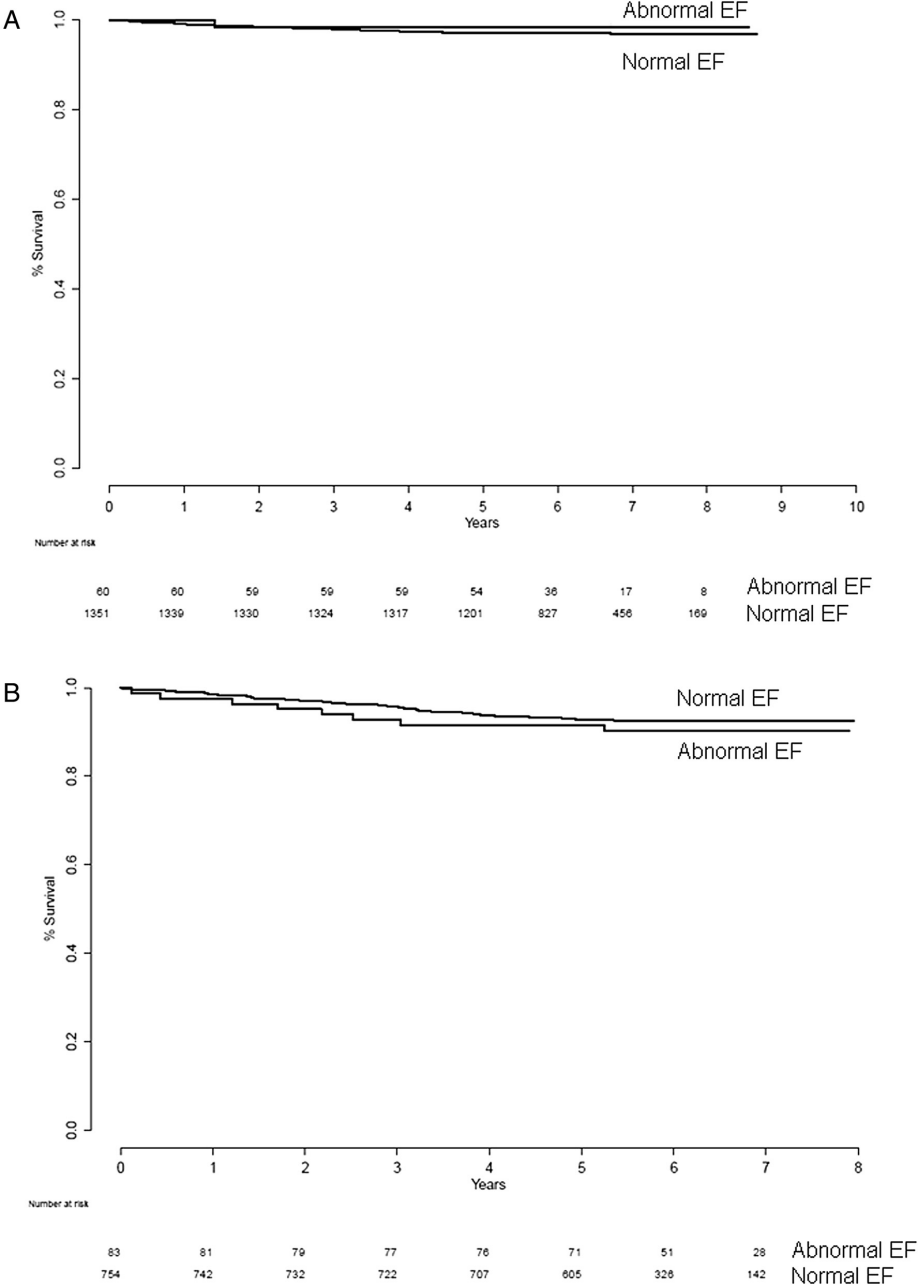
Survival for normal EF vs. abnormal EF for normal stress-only (A) and normal stress-rest (B) patients.

**Figure 3:**
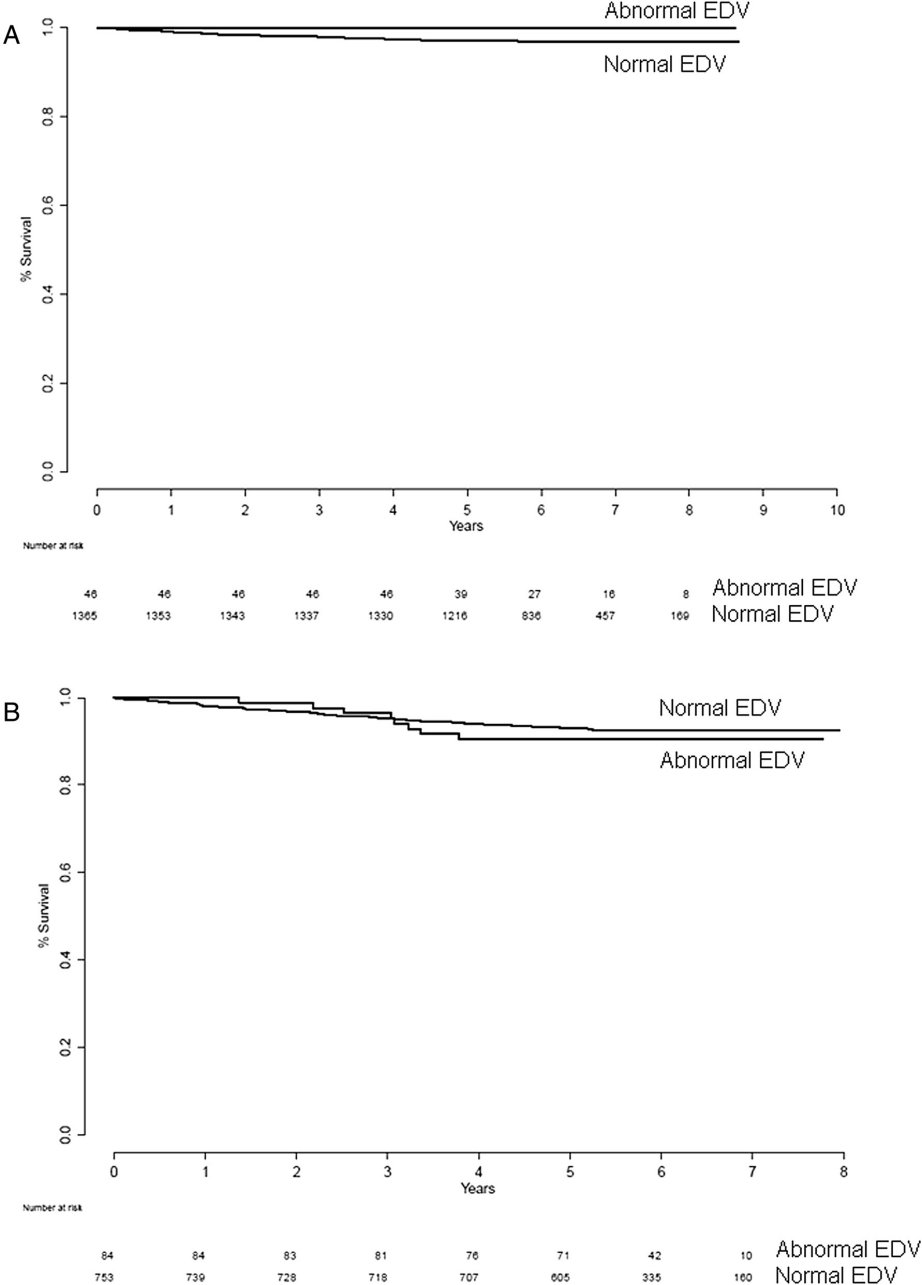
Survival for normal EDV vs. abnormal EDV for normal stress-only (A) and normal stress-rest (B) patients.

**Figure 4:**
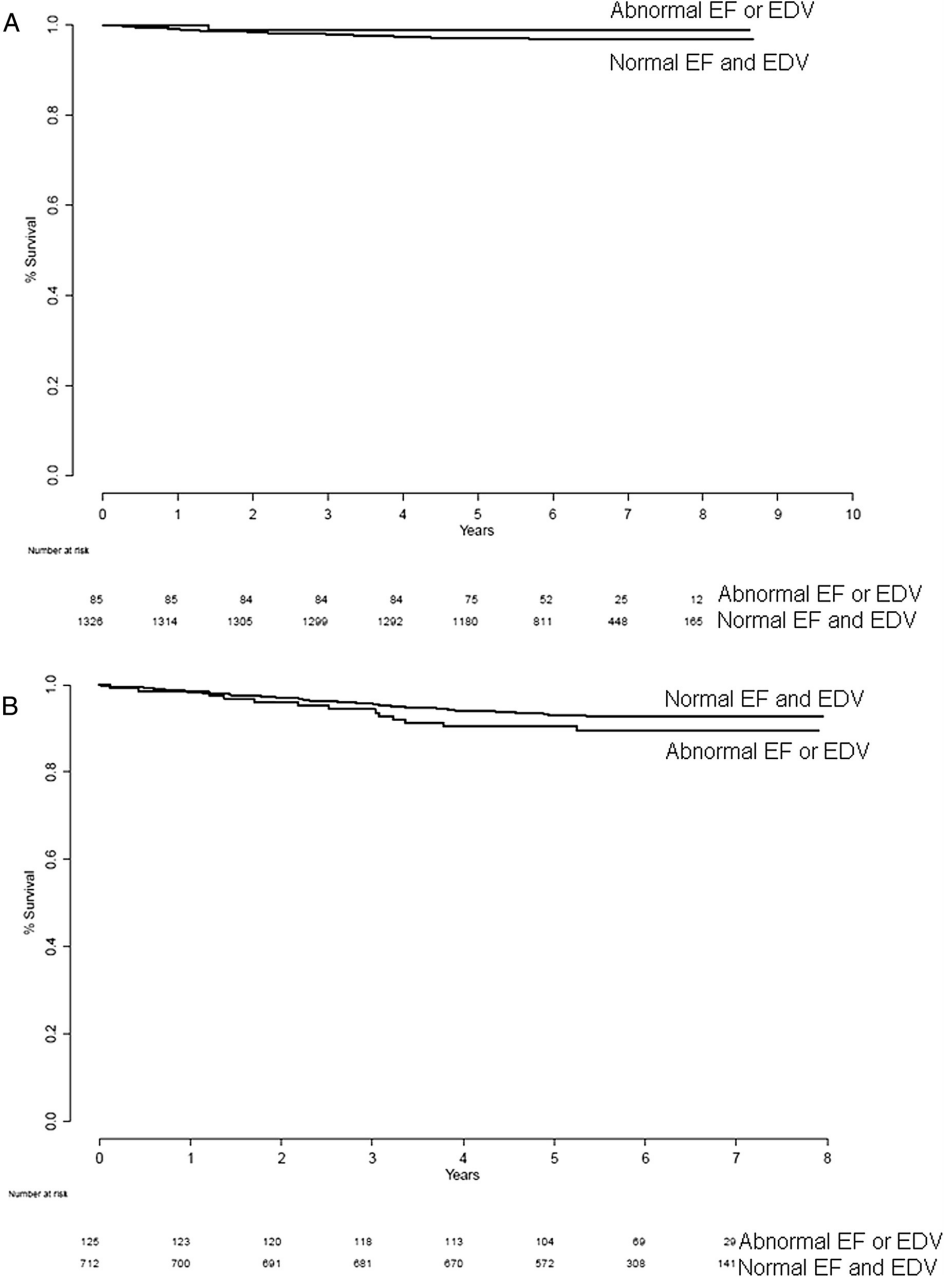
Normal EF and EDV vs. abnormal EF/EDV for normal stress-only (A) and stress-rest (B) patients.

Table 2 shows the number of patients with missing EF or EDV (due to difficulties in gating specific patients or patients examined before September 2004; see above), which was excluded in the subsequent analyses. Table 1 shows the number of patients, number of events, and annual event rate for the different groups. No statistically significant differences in event rate between patients with normal and abnormal EF in the normal stress-only group (*p* = 0.55) or in the normal stress-rest groups (*p* = 0.23) were found (Figure 2). When regarding normal vs. abnormal EDV in the normal stress-only group, no significance in event rate (*p* = 0.32) was found. Neither was there any statistically significant difference in event rate between patients with normal and abnormal EDV in the normal stress-rest groups (*p* = 0.47; Figure 3). When regarding patients with normal EF and EDV vs. patients with abnormal EF or EDV, no statistically significant differences in event rates were found (*p* = 0.52 for normal stress-only patients and *p* = 0.21 for normal stress-rest patients; Figure 4).

**Table 2:** Number of patients with missing EF or EDV

	**Normal stress-only group**	**Normal stress-rest group**
Missing EF (all)	*525*	*325*
Missing EF (men)	217	190
Missing EF (women)	308	135
Missing EDV (all)	*804*	*374*
Missing EDV (men)	311	207
Missing EDV (women)	493	167

### Discussion

The present study demonstrated cardiac mortality and cardiac events over a mean 6.2-year follow-up period in 3,432 patients who showed a normal MPS in the study. We found that the event rate was significantly lower in normal stress-only patients compared to normal stress-rest patients and non-normal patients. Regarding LV function data, we did not find any significant difference in event rate between normal vs. abnormal EF, normal vs. abnormal EDV or normal EF, and EDV vs. abnormal EF or EDV for either the normal stress-only patients or the normal stress-rest patients. To the best of our knowledge, this is the first study comparing normal and abnormal EF and EDV in normal stress-only and normal stress-rest patients. In theory, the prognosis should be worse for patients with abnormal EF and/or EDV. In the present study, surprisingly, the event rate was lower for the patients with abnormal EF and/or EDV compared to those with normal EF/EDV for both normal stress-only and normal stress-rest patients, but it was not statistically significant. This could be due to the small abnormal study groups. If the study groups were larger, the results might have been different. Further studies are needed to evaluate this.

There could be different reasons for the difference in event rates between the normal stress-only and the normal stress-rest groups. It is well known that the perfusion data in MPS could appear normal in patients with balanced three-vessel disease and in patients with subendocardial infarction. Some of these patients, who have worse prognosis than individuals without ischemic heart disease, could be present in the stress-rest group. It is also known [14] that the cardiac event rate is higher with pharmacologic stress MPS than with exercise MPS for both normal and abnormal MPS results. In this study, the proportion of exercise tests was higher in the stress-only group compared to the normal stress-rest group, which might be an explanation for the slightly better prognosis in the normal stress-only group.

Some previous studies have been published addressing outcome in stress-only MPS studies. The largest study included 16,854 patients, and it was concluded that patients determined to have a normal MPS on the basis of stress imaging alone had a similar mortality rate as those who had a normal MPS on the basis of evaluation of both stress and rest images [7]. However, their end point was overall mortality, not cardiac events. Duvall et al. [15] investigated the prognosis of normal stress-only MPS studies compared to normal stress-rest MPS in order to establish its effectiveness in an emergency department setting. They found that a normal stress-only MPS study had a benign 1-year prognosis similar to a stress-rest study when performed in the emergency department. Only low-intermediate risk patients were included in their study, and the end point was all caused by mortality. Other studies, with end points of fatal and non-fatal cardiac events, have reported similar results, both for ^201^Tl and ^99m^Tc [6,16-18]. The present study has a longer follow-up period (mean, 6.2 years) than the previous studies (mean follow-up between 2 and 4 years). Mathur et al. [19] found that stress-only imaging with attenuation correction in symptomatic patients is an efficient method which appropriately identifies at-risk and low-risk patients, yielding a low percentage requiring rest imaging. A previous study from our group also demonstrated that adding attenuation correction stress-only images to non-attenuation-corrected stress-only images reduced the need for unnecessary rest studies substantially [20].

Most MPS studies on prognostic information have used summed stress and rest scores in order to categorize studies as normal or abnormal. This approach has both advantages and disadvantages. The advantages are that the categorization can be done in a standardized way that is easy to describe and easy to reproduce by others. It does not, however, reflect the real interpretation situation where physicians consider more information than only summed stress and rest scores. In our study, we used the final report to categorize studies as normal or abnormal. We have therefore shown that benign prognoses for normal stress-only patients, previously reported by others, also are true in clinical routine.

### Study limitations

Since this study was retrospective, we could not obtain reliable information about risk factors such as diabetes, previous coronary artery disease, smoking habits, and hypertension. We also did not have any information if the diagnosis of normal/non-normal study was confirmed by other diagnostic tests.

Also, if the patient has a high risk of or known ischemic heart disease, the physician in charge of the study is probably more likely to decide that a rest study should be performed, if he/she is slightly doubtful when assessing the stress images, than if the patient has a low risk of ischemic heart disease. This could create a bias in the data and could explain the somewhat poorer outcome in the normal stress-rest group compared to the normal stress-only group.

## Conclusions

Patients with a normal stress-only study had an excellent prognosis over a mean follow-up time of 6 years. Thus, omitting the rest study if the stress study is normal is a safe procedure.

## Competing interests

LE and MO are stockholders of EXINI Diagnostics AB. ET has no competing interests.

## Authors’ contributions

LE and ET designed the study and drafted the manuscript. MO performed the statistical analysis and helped draft the manuscript. All authors read and approved the final manuscript.
